# Speakers and Speech

**Published:** 1888-06-02

**Authors:** 


### The Hospital

A Weekly Institutional Journal of

**Science, Medicine, Nursing, and Philanthropy.**

*For Hospitals, Asylums, Medical Practitioners, Students, Nurses, Families, Parishes, Congregations, and the Charitable Public.*

Vol. IV. No. 88.] [June 2, 1888.

### Speakers and Speech.

It must be a serious task to many public speakers and preachers to prepare a speech or a sermon on hospitals. The topic is annual, and many people think it is worn threadbare. It has, besides, the peculiar quality of presenting so obviously convincing a case that it seems like pointing a denizen of Regent Street the way to Pall Mall to say anything at all about it. But those who are members of a household know much better the characters of the husband and wife and the general current of domestic affairs than those who merely live in the same square.

The circumstances of hospitals are not happy. The managers of the various firms, to use a business metaphor, do not lie on beds of roses. They have to face the problem of a great and growing disparity between the numbers of those who urgently need hospital relief and the resources available for their help. Those managers, be it remembered, have no personal financial interest in hospitals; their concern for them is of a purely benevolent and disinterested character. But though that is so, they feel in most cases almost as deep an anxiety about the business of the hospitals as they do about their own individual cares. Responsible managers are not happy because they recognise that a crisis has arrived in the history of their institutions. It is not a crisis of a merely local or temporary character; if it were it might be dealt with by a single resolute effort. It is a time of enforced halt, of looking round to all quarters to see what is likely to happen, and what may be the wisest, or perhaps the only possible course for the future.

The one thing above all others which the friends of hospitals are striving at the present moment to secure is—the serious attention of intelligent and right-hearted men. If they might choose they would particularly lay hold of the minds of clergymen, members of Parliament, and all who influence the people by public speech. The two classes named have it in their power to rebuild the shaking walls of the medical charities and to infuse new life into their enfeebled organisations. What could those classes accomplish if they were sufficiently resolved? They could convince the whole community that it is every individual person's duty to help hospitals according to his measure. That single conviction, firmly rooted in each man's and woman's heart, would make all *bonâ fide* hospital work secure and prosperous, at least during the lifetime of the present generation.

But in order to produce conviction in another's mind a man must be first convinced himself. Are preachers and members of Parliament convinced, in any deep and earnest sense, of the untold value of hospitals and of the absolute necessity there is for making an effort to place them on a new and wider basis of support? On a basis indeed co-extensive with the whole population? If they are not, they should at once put themselves in the way of being convinced; for that is the one result now wanted above all others. The present, we admit, is an age of feeble convictions, of shrugging of the intellectual and moral shoulders, of questioning and uncertainty, of pessimism and despicable *laissez faire*. But there are certain institutions which do not admit of doubt, about which the most absolute dogmatism is not only admissible, but commendable; institutions which to pass by with cold neglect does not prove that too much intellectual culture has produced a justifiable indifference, but that too much self-indulgence and too little sense of duty have resulted in a want of intellectual capacity for understanding the difference between worthy and unworthy objects, between that which ought to excite our utmost energy and enthusiasm and that which it may be well to leave alone.

It may be asked—why should hospitals be in such a critical condition at this particular time? The answer is obvious and conclusive—because the population has grown by leaps and bounds, and the sick poor have increased in proportion; whilst the area from which hospitals receive their main support has hardly been extended at all. In former periods a few rich people supported medical charities almost entirely, and the bulk of the population recognised neither the duty nor the necessity for helping them even a little. This is the case still to a very surprising extent. Hardly any effort is made by the general public beyond the single annual collection taken on Hospital Sunday, and the cartloads of pence collected with so much painstaking zeal by the Hospital Saturday Fund. But will any reasonable man call these single annual collections adequate efforts on the part of a great nation to support its voluntary hospital system?

Hospital managers, as has been said, feel that a crisis has arrived. The principal efforts they now put forth are made with the object of convincing the public of this fact. This journal has, as its chief reason for existence, the enlightenment and stimulation of the people in the matter of hospitals, their good work, their deep necessity, and their absolute and incalculable value to the public at large. The tables we publish elsewhere will furnish ample evidence of the work they are now doing, and of the straits to which they are reduced. The natural appeal of managers at the present time, and of this journal, as the mouthpiece of hospitals throughout the United Kingdom, is to clergymen and members of Parliament, because their position gives them unique opportunities of influencing general opinion. Our desire is not merely to secure a liberal collection from every place of worship on Hospital Sunday and to raise one hundred thousand pounds in London alone, but to have the whole question of hospitals, their noble work, their constant needs, their scientific and practical services to the community, and all their other claims so placed before discriminating audiences that each and all may be convinced that a duty is owed to those institutions which nothing but personal thought and personal service can pay. We wish the public to be made clearly to understand that the time for thought and action is now. If, as the result of the Hospital Sunday efforts of the present year, a hundred thousand pounds were raised, and a hundred thousand people in London and ten times that number in the country, who had before been indifferent or lukewarm, could be converted into active and intelligent workers on behalf of hospitals, the triumphant and enduring success of the voluntary system of medical charities would be permanently secured.

Great is the power of convincing speech. By it battles have been lost and victories have been won; peoples have been regenerated and nations have been destroyed; governments have been established and power has been shattered to its fall; old religions have been swept away and purer faiths established in their place. It is this great and noble gift of human speech which we desire to enlist on behalf of hospitals. What worthier theme could the most benevolent desire; what more generous inspiration could be invoked by the most eloquent tongue? History furnishes no records of diviner deeds than those done in hospitals for the poor, the sorrowing, and the sick. It is to make such deeds permanent and universal that we ask all those whom opportunity serves to rise to the greatness of the great occasion and to resolve to polish and lay at least one stone in that temple of charity which we would fain make strong, beautiful, and enduring for all future time.

